# Beneficial effect of traditional Chinese medicine fumigation “Bone-healing Powder” in postoperative pain and recovery of neurological function of traumatic thoracolumbar spine fractures: A case-control study

**DOI:** 10.1097/MD.0000000000011983

**Published:** 2018-08-21

**Authors:** Xiu-Li Wang, Xiu-Ping Zhu, Dong-Xing Ji, Jun Wang, Rui-Hua Zhai, Ping Li, Xue-Fei Yang

**Affiliations:** a Department of Outpatient; b Department of Pediatrics; c Department of Orthopedics, Yidu Central Hospital of Weifang; d Department of Orthopedics, Qingzhou Municipal Hospital, Qingzhou; e Department of Ear-Nose-Throat (ENT), Yidu Central Hospital of Weifang; f Department of Thoracic Surgery, Weifang People's Hospital, Weifang, P.R. China.

**Keywords:** bone-healing powder, neurological function, pain, swelling, thoracolumbar spine fractures, traditional Chinese medicine fumigation

## Abstract

**Background:**

Thoracolumbar spine (TLS) fractures are commonly associated with the young healthy population, with its risk factors including both high-energy traumas and neurological deficit. The underlying mechanisms of traditional Chinese medicine (TCM) and TLS fractures have been explored. Therefore, our prospective study was conducted in order to explore the beneficial effects of TCM fumigation “Bone-healing Powder” method in both postoperative pain as well as the recovery of the patient's neurological function following healing from their traumatic TLS fractures.

**Methods:**

Patients dealing with traumatic TLS fractures were randomly assigned into both the control and the intervention groups based on whether or not they received prior TCM fumigation in addition to any and all conventional therapy. Imaging indexes, including height of the injured vertebra (%), Cobb angle (°), horizontal displacement (%), compression area (%), sagittal diameter (%), and degree of both the swelling and pain regarding the fractures were observed and recorded both before and after the treatment for proper progression documentation. The neurological function was evaluated according to American Spinal Injury Association (ASIA) classification in order to investigate whether TCM fumigation “Bone-healing Powder” could affect the recovery of the patient's neurological function.

**Results:**

Following the treatment as well as 1 year after its completion, patients who received TCM fumigation presented a higher height of their previously injured vertebra (%) and sagittal diameter (%), while a lower Cobb angle (°), horizontal displacement (%), and compression area (%) than those who were part of the conventional therapy group. A week posttreatment, patients that received TCM fumigation also showed no signs of swelling or mild pain. One year following the treatment, patients receiving TCM fumigation demonstrated an improved neurological function.

**Conclusion:**

These findings help to indicate that TCM fumigation “Bone-healing Powder” reduces the degrees of postoperative pain and swelling, and effectively improves recovery of the neurological function of those patients with traumatic TLS fractures, proving its worth as a clinical method in treatment.

## 1 Introduction

Traumatic spinal cord injury (SCI) is a major contributor to neuron death as well as axonal damage leading to both functional motor and sensory loss, presenting limited regeneration due to the adverse microenvironment which includes both neuro-inflammation and glial scarring.^[1]^ Thoracolumbar spine (TLS) fractures are common injuries following an encounter of blunt trauma including a significant morbidity often accompanied with a neurological deficit and characterized by a damaged posterior vertebral body wall.^[2]^ Besides, anterior transthoracic decompression and fusion is associated with better recovery of neurological function.^[3,4]^ As for the surgical treatment of a TLS fracture, combining simplified percutaneous external pedicular fixation and intracorporeal bone grafting methods have been discovered as being feasible and effective.^[5]^ However, since TLS fracture patients with surgical treatment suffered more pain, it is very important to handle the postoperative situation, especially pain and neurological function.^[6]^ Fortunately, the traditional Chinese medicine (TCM) treatment is an effective way when treating osteoporosis with a relatively less significant side-effect,^[7]^ making TCM a promising choice for the treatment of traumatic TLS fractures.

TCM has been applied in order to prevent, treat, and cure various diseases for centuries.^[8]^ It was found by Pang et al^[9]^ that Berberine, a classical natural medicine, was central to the treatment of type 2 diabetes by improving insulin resistance as well as inhibiting the gluconeogenesis in liver. A brown seaweed, Sargassum, has also been applied in the treatment of thyroid diseases such as Goitre and Hashimoto thyroiditis.^[10]^ TCM treatment was already reported as being beneficial for the alleviation of pain as well as speeding up bone healing in proximal humeral fracture.^[11]^ TCM is able to alleviate osteoporosis, which has features such as low bone mass, the loss of bone microstructure, and higher rate of bone fracture.^[12]^ The main components of the “Bone-healing Powder” fumigation include safflower, Angelica Sinensis, Radix Dipsaci, and Rhizoma Drynariae. For example, Weishi Bitong Xifang fumigation was an efficacious and safe option for the treatment of knee osteoarthritis.^[13]^ Angelica Sinensis, one of the herbs screened out by the suppression of nuclear factor kappa B luciferase activity, has been confirmed to exert anti-inflammatory effects over lipopolysaccharide plus interferon-γ-stimulated peritoneal macrophages by decreasing the nitric oxide production.^[14]^ Rhizoma Drynariae is a classic TCM with a long history of safe use for the treatment of bone fractures and joint diseases whose roots have been reported to have therapeutic effects on osteoporosis and bone fracture in many studies.^[15]^ The rhizomes of Radix Dipsaci have long been applied as an anti-osteoporosis and tonic agent in TCM for the therapy of low back pain, traumatic hematoma, and bone fractures thanks to its roots having therapeutic effects on osteoporosis.^[16]^ Additionally, TCM has offered many alternative dosage forms, such as an herbal bath formula, meant to alleviate both pain and swelling.^[17]^ Also, TCM, especially all kinds of herbs involved in the practice, is known to be effective in improving peripheral nerve regeneration, making it a good choice for the postoperative treatment.^[18]^ Based on the aforementioned information, suggestions have been made that TCM can make great differences in fractures, degrees of pain, and swelling following fractures. Therefore, we aim to evaluate the effect TCM fumigation has on the postoperative pain as well as the recovery of neurological function of SCI in TLS fractures through observations made of the height of injured vertebra (%), Cobb angle (°), horizontal displacement (%), compression area (%), sagittal diameter (%), degrees of swelling and pain, as well as the neurological function.

## 2 Materials and methods

### 2.1 Ethics statement

The study was approved by the Ethics Committee of Weifang People's Hospital with all patients signing the informed consent.

### 2.2 Study subjects

A total of 282 TLS fractures patients treated in Orthopedics of Weifang People's Hospital for 2 years from January 2013 to January 2015 were selected to take part in our prospective study. According to the random number table method, 282 patients were divided into both the intervention group and the control group (each 141 cases). A double-blind method was employed in order to make the treatment and efficacy evaluation. Inclusion criteria included: patients meeting the diagnostic criteria according to Guiding principles of clinical observation on the treatment of traumatic fractures with new Chinese medicine; patients agreeing to sign informed consent; patients confirmed as TLS fractures by both frontal and lateral x-ray images, computed tomography (CT), or magnetic resonance imaging (MRI). Exclusion criteria included: patients had severe diseases of important organs and systems (liver, kidney, digestive tract, immunity, blood, endocrine, and metabolism); patients had pathological fractures; patients had neurological injury.

### 2.3 Surgical procedures

Patients were anesthetized via inhalational anesthesia following tracheal intubation or epidural anesthesia while in a prone position. A fracture vertebra was then aligned with waist bridge. The chest and anterior superior iliac spines (ASIS) on both sides were blocked up and the abdomen was suspended. Vertical pressure was then put on the fracture of the patient's back vertebra and manual reduction was conducted. Then, we observed a lateral fluoroscopy in order to further determine the shape of the injured vertebral body as well as the angle of the screw. The screws were then screwed into the injured vertebral body along with the normal vertebrae adjacent to the injured endplate according to the conventional entrance point provided by the posterior median approach. Direction of the screw entrance was coronal deviation 15° to 20° (angle based on preoperative CT measurements). In the median sagittal section, the normal vertebrae was parallel to that of the vertebral body, and the injured vertebrae screws were tilted from 5° to 10° in the direction point of the normal endplate. After screws were entered successfully, postural reduction and distraction reduction were successively performed. With evidence of nerve injury symptoms, both inter-laminar fenestration and decompression methods were performed, and the nerve roots and dura sac were gently pulled out to the opposite side. The bone block was subsequently reduced when the bone fragments protruding from a vertebral fracture was determined. Finally, an articular cartilage was excised and a bone groove approximately 0.5 cm (width) × 1.0 cm (depth) was cut and removed. A few bone pieces were also cut out from the adjacent lamina and spinous processes, while implanting into the intervertebral joint, with the lumbar zygapophysial joint fusion performed at last. A drainage tube was then placed routinely and the wound was closed. Postoperative routine prevention of infection was conducted, and hemostatic drugs were used. Patients who were also dealing with a spinal cord nerve injury received dehydration, hormone, and neurotrophic drugs treatment. After about 24 to 48 hours, the drainage tube was pulled out, and the thread was also removed after 12 days. After 3 weeks, the patients could walk with the assistance of a brace until 3 months after the operation had passed, with excessive bending to be avoided during the period.

### 2.4 Grouping

Two groups of patients received basically same anesthesia and surgical treatment. Following the operation, the control group was treated with a conventional therapy, namely postoperative routine prevention of infection was conducted, with use of certain hemostatic drugs. Patients who were also dealing with a spinal cord nerve injury received dehydration, hormone, and neurotrophic drugs treatment. However, patients involved in the intervention group were treated with a TCM fumigation, in addition to the conventional therapy. TCM was put into special cloth bags, heated, and insulated to fumigate in the factures after boiling to sterilize. Simultaneously, patients were treated with the corresponding functional training.

TCM fumigation was conducted through the following processes: all herbs were placed into the pot, added along with up to 3000 to 4000 mL of water, soaked for 30 minutes, and then boiled over mild heat for 30 minutes. The TCM liquid obtained from this fumigation was then stored for further use. The patient would go on to sleep on the fumigation bed with a piece of protective pad sheet under the affected area. The TCM fumigation instrument was then connected to the power supply after addition of the liquid, and the spray head was directed at the affected area for further fumigation. The spray head should be held approximately 30 to 40 cm away from the affected area in order to prevent the skin from experiencing scalding. We sprayed the area for 30 minutes and let the patient rest afterwards. The treatment was conducted twice a day for 10 days as the course of treatment lasted between 3 and 5 courses. During the fumigation, the room should keep in warmth with closed doors and windows at 22 °C or 24 °C. The exposed limbs should be covered. After treatment, dry towel was used to dry the water drop on the affected limb. Patients should put on clothes in order to prevent catching a cold or succumbing to other conditions. The distances between both the spray head and the affected limb were adjusted according to the patients’ ages, and the temperature tolerance of the skin to reach the appropriate distance, generally ranging between 30 and 40 cm. If the distance appeared to be too close, the skin would be scalded. If the distance was too far, the efficacy would be affected. In the course of TCM fumigation, the inspection must be strengthened. Considering the high temperature of the affected limbs and room temperature that would increase the sweating of the patients, the patients were instructed to replenish water in time and to drink some light salt water or juice properly before treatment to avoid going into shock and syncope. If local skin reactions, such as pale, erythema, blisters, itchy pain arose, fumigation should cease immediately and the doctor should be informed to deal with these responses. Regular sanitary practices should be observed such as pieces of sheet needing replacement in time in order to eliminate any cross-infection.

### 2.5 Endpoints and indicators

The main endpoint of the experiment was the proportion of patients who graded D to patients who graded E according to the results provided by the American Spinal Injury Association (ASIA) Impairment Scale 1 year posttreatment.

The secondary endpoints were: 1, Proportion of patients with swelling to those without swelling at the 7th day after treatment; 2, Adverse events during the trial from the signing of the informed consent were recorded.

### 2.6 Imaging measurement indexes of spinal fracture

Before treatment, the 7th day following treatment, and 1 year after the treatment, an x-ray plain film (using Kodak 2000 DR system (Eastman Kodak Corp., Rochester, MN), frontal, and lateral side, automatic exposure) in both the control and intervention groups was applied in order to measure the height of the injured vertebra, Cobb angle (the angle formed by 2 intersecting lines drawn on the upper and lower margin of the injured vertebral body in the multisice computed tomography [MSCT] sagittal reconstruction images), and the horizontal displacement of sagittal plane. CT was used in order to measure vertebral compression area and sagittal diameter.

### 2.7 Degree of limb swelling

According to the grading criteria of soft tissue injury,^[19]^ the degree of limb swelling was divided into 4 grades. Those 4 grades went with the following classifications: no swelling, mild swelling, moderate swelling, and severe swelling. Mild swelling refers to normal skin while also exhibiting a little tightness and shallow dermatoglyph. Moderate swelling is more obvious in appearance and with a disappeared dermatoglyph; there is an increased temperature on the skins surface and no blisters. Severe swelling means tight skin and noticeable blisters. Comparisons of degree of limb swelling were made before treatment as well as on both the 1st and 7th days after treatment.

### 2.8 Degree of limb pain

The visual analogue scale (VAS) was used in order to assess the degree of pain.^[20]^ Patients were asked to underline on a straight line with 10 scales. The visual analogue scale scoring went as follows: 0 means no pain, 10 for unbearable pain, 1 to 3 for mild pain, 4 to 6 for moderate pain, and 7 to 9 for severe pain. Comparisons of degree of limb pain were made before treatment as well as on the1st and the 7th day's posttreatment.

### 2.9 Neurological evaluation

The neurological function was evaluated by adopting the ASIA classification (Table 1) both before and on the 7th day following the treatment, as well as 1 year after treatment.^[21]^

**Table 1:**
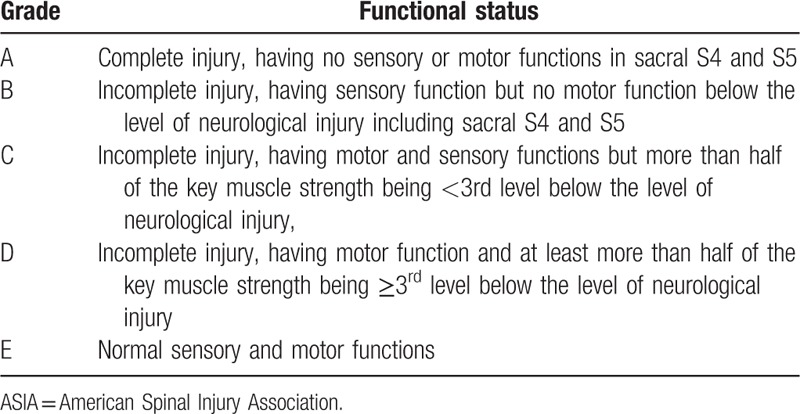
ASIA neurological function classification.

### 2.10 Sample size calculation

The calculation of the sample size was predominantly based on the main therapeutic indicators. This study assumed that the clinical efficacy of the intervention group was superior to when we compare with that of the control group, and approximately 282 subjects were assigned to both the control group and the intervention group using the ratio of 1:1. According to Fisher exact probability method, the statistical level of significance was set at “*α* < 0.05 (two-tailed).” In order to determine that the clinical efficacy of the intervention group was better than that found with the control group, 90 cases could provide 90% assurance. According to the drop-off rate of 20%, each group needed at least 120 subjects. A total of 282 subjects were included in this paper, which reached the sample size of statistical hypothesis.

### 2.11 Statistical analysis

Data were analyzed using the SPSS software version 21.0 (IBM Corp. Armonk, NY). Measurement data were expressed using the mean ± standard deviation and further analyzed using a *t* test. Enumeration data were thoroughly expressed as a percentage or rate and further validated using chi-square test. Ranking data were measured using the rank sum test. Comparisons between groups were made and analyzed also using a *t* test. Multiple groups were compared by employing the one-way analysis of variance (ANOVA). On the basis of statistical significance, *t* test of pairwise comparison was carried out. *P* < .05 was considered as being statistically significant.

## 3 Results

### 3.1 Baseline characteristics of patients in the intervention group and the control group

A total of 282 TLS fractures patients were enrolled to take part in this study. There were 125 women and 157 men, with their ages ranging between 13 and 70 years old, and their mean age at 37.4 ± 10.2 years. Among the subjects, 133 cases were presented as left TLS fractures patients and 149 cases were presented as right TLS fractures patients. According to the Sehatzker classification,^[22]^ there were 42 cases of type I, 98 cases of type II, 85 cases of type III, and 57 cases of type IV. The reasons for injuries were high falling injuries (119 patients) and traffic injuries (163 patients). There were 104 patients receiving treatment on the 1st day after injury, 124 patients receiving treatment 2 or 3 days following the injury, and 54 patients receiving treatment >3 days following injury. There was no statistical significance detected between the 2 groups in age, sex, and cause of injury (all *P* > .05). There were no adverse events associated with the drug “Bone-healing Powder” in the study, proving that the treatment was safe, with good tolerability.

### 3.2 Patients received TCM fumigation present with better imaging measurement indexes

**Figure 1:**
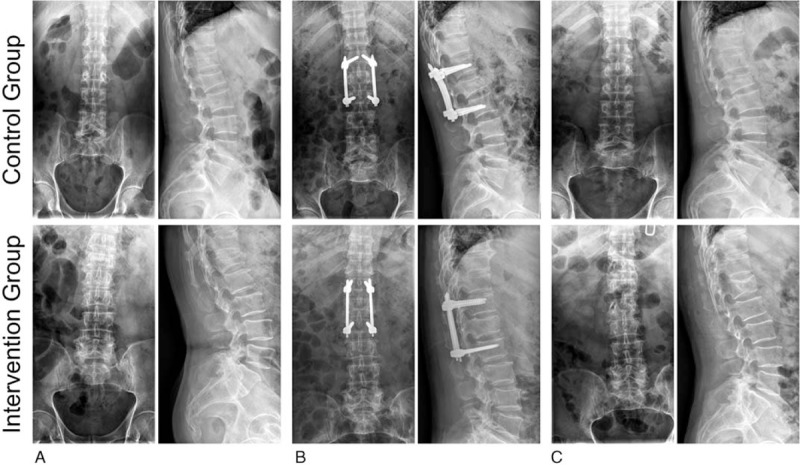
X-ray imaging before and after treatment of 2 patients in the intervention and control groups. The control group shows the first lumbar vertebra compression fracture, before and after treatment x-ray imaging data, postoperative recovery is not good. The intervention group shows the first lumbar vertebra compression fracture, before and after treatment x-ray imaging data, postoperative recovery is good. A, Before treatment; B, 7 days after treatment; C, 1 year after treatment.

Before treatment, in comparison with the control group, the intervention group showed no significant differences in the imaging measurement indexes, which included height of injured vertebra (%), Cobb angle (°), horizontal displacement (%), compression area (%), and sagittal diameter (%) (all *P* > .05). In the intervention group, imaging measurement indexes immediately after treatment and 1 year after treatment showed a significant improvement when compared with the indexes prior to the treatment. The height of injured vertebra (%) and sagittal diameter (%) exhibited a significant increase, while Cobb angle (°), horizontal displacement (%), and compression area (%) all showed significant decreases (all *P* < .05). Index changes in the control group immediately after treatment and 1 year after treatment had not shown any statistical significances in comparison to those findings prior to the treatment (all *P* > .05). The height of the injured vertebra (%) and sagittal diameter (%) both slightly increased, while Cobb angle (°), horizontal displacement (%), and compression area (%) slightly decreased. Immediately after treatment and 1 year after treatment, in comparison with the control group, the intervention group presented with a higher height of injured vertebra (%) and sagittal diameter (%), in addition to a lower Cobb angle (°), horizontal displacement (%), and compression area (%) (all *P* < .05) (Table 2 and Fig. 1). Taken together, patients received TCM fumigation presented with better imaging measurement indexes.

**Table 2:**
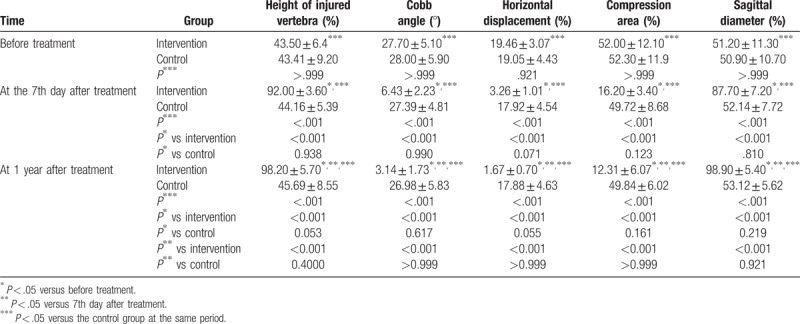
Comparisons of height of injured vertebra (%), Cobb angle (°), horizontal displacement (%), compression area (%), and sagittal diameter (%) in the control and the Intervention groups before treatment, at the 7th day after treatment and 1 year after treatment.

### 3.3 Patients received “Bone-healing Powder” fumigation have improved postoperative swelling and pain of traumatic TLS fractures

Before treatment and 1 day after the treatment, there were no significant differences in the distribution proportions of both the degrees of swelling and pain between the intervention and the control groups (all *P* > .05). Seven days posttreatment, no swelling patients and mild pain patients were obviously more in the intervention group than in the control group (*P* < .05), and no patient showed severe swelling in the intervention group. The results indicated that the recovery of swelling and pain was noticeably better in the intervention group than in the control group (Table 3).

**Table 3:**
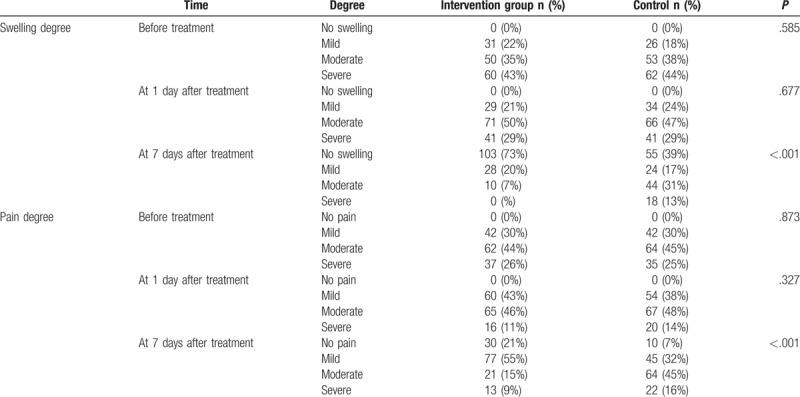
Comparisons of degrees of swelling and pain of patients in the control and intervention groups.

### 3.4 Patients treated with “Bone-healing Powder” fumigation show improved postoperative recovery of neurological function of traumatic TLS fractures

Before treatment, there were no significant differences in the ASIA classification between the intervention group and the control group (*P* > .05), as well as presenting no E-level patients. After the treatment, the proportion of C-level to D-level patients was slightly higher in the intervention group than in the control group (*P* > .05). One year following the treatment, the neurological function of patients in the intervention group (except A-level patients) improved their ASIA classification more than one grade higher. Moreover, there were more D and E-level patients in the intervention group compared with the control group (all *P* < .05). These results would go on to show that the recovery of neurological function was better in the intervention group than in the control group (Table 4).

**Table 4:**
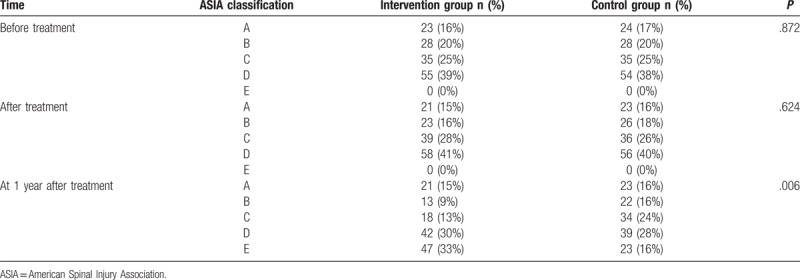
ASIA classification of patients between the intervention group and the control group.

## 4 Discussion

There are an increasing number of patients suffering from postoperative pain due to orthopedic surgery and bone fracture worldwide.^[23]^ This study investigated the beneficial effects of employing a TCM fumigation method known as “Bone-healing Powder” in both the postoperative pain and recovery of neurological function of traumatic TLS fractures. It was also discovered that TCM fumigation “Bone-healing Powder” reduced the degree of both pain and swelling, and effectively improved the recovery of neurological function in patients with traumatic TLS fractures.

Initially, patients who received TCM fumigation demonstrated a taller height of injured vertebra (%) and sagittal diameter (%), while presenting a lower Cobb angle (°), horizontal displacement (%), and compression area (%) than those that only received a conventional therapy routine. The injured vertebral height and Cobb angle show obvious improvement after operation.^[24]^ The sagittal vertebral canal diameter had been remarkably enlarged when para-spinal-approach reduction and fixation were used in order to treat Denis B type thoracolumbar burst fractures with deficiencies of neurological function.^[25]^ Specifically, a previous study showed that TCM treatment could promote spinal movement and exercise as well as decreasing the Cobb angle, which is dually a simpler and more cost effective route than conventional treatment.^[26]^

Additionally, our data made further suggestions that the TCM fumigation “Bone-healing Powder” method could reduce both pain and swelling after the traumatic TLS fractures operation, consistent with previous findings that TCM was beneficial in treating distal radius fracture.^[27]^ It has often been reported that surgical treatment of the fractures was often accompanied by pain and swelling after operation, which exerted great influence on patients’ quality of life.^[28]^ Interestingly enough, TCM has been applied in an attempt to reduce pain and swelling following the surgical treatment, for instance, Shuang-Qi gout capsule, a TCM prescription, has been used to treat joint pain, inflammation, and gout arthritis, Huangcaowu, a folk herbal medicine, has been used to alleviate both allodynia and swelling, and CDNR, a Chinese herbal paste, was used in order to promote fracture healing, which not only facilitated the growth of bone cells but also added to the biomechanical strength of the healing bone.^[29–31]^

Moreover, our study made further revelations that TCM fumigation “Bone-healing Powder” was helpful when studying the recovery of neurological function after the traumatic TLS fractures operation. For example, radial neurological injury occurred after a double-plate fixation regarding the treatment of intercondylar humeral fractures and caused both a sensory and motor deficit.^[32]^ In order to combat that, the TCM formula was helpful in promoting the peripheral nerve regeneration, fortunately with little side effects, with the mechanisms also being related to its pharmacological effect of enhancing proliferation, differentiation, and migration of neural stem cells, as well as regulating neural stem cell microenvironment,^[33,34]^ and promoting nerve growth factor expression.^[35]^ It was also suggested that TCM Safranal was protective for neurological functions in spinal cord injury, which was associated with its anti-apoptotic, anti-inflammatory, and edema-attenuating effects.^[36]^ All of these remedies and methods can provide a potential mechanism for TCM in treating the postoperative recovery of neurological function after traumatic TLS fracture.

In conclusion, the present study provided evidence that TCM fumigation “Bone-healing Powder” reduced the degrees of postoperative pain and swelling, while improving the recovery of neurological function of traumatic TLS fractures. We attempted and seemingly succeeded in providing a new approach for the treatment of postoperative pain as well as neurological function recovery of traumatic TLS fractures. However, the number of cases and the duration of follow-up were limited, thus making it necessary for us to collect further cases and experiments.

## Acknowledgments

The authors would like to give our sincere appreciation to the reviewers for their helpful comments on this article.

## Author contributions

**Conceptualization:** Xiu-Li Wang.

**Data curation:** Xiu-Ping Zhu, Dong-Xing Ji.

**Formal analysis:** Xiu-Ping Zhu, Dong-Xing Ji.

**Investigation:** Jun Wang.

**Methodology:** Rui-Hua Zhai.

**Resources:** Rui-Hua Zhai.

**Supervision:** Ping Li.

**Validation:** Ping Li.

**Visualization:** Xue-Fei Yang.

**Writing – original draft:** Ping Li, Xue-Fei Yang.

**Writing – review & editing:** Ping Li, Xue-Fei Yang.
